# Urban-rural differences in catastrophic health expenditure among households with chronic non-communicable disease patients: evidence from China family panel studies

**DOI:** 10.1186/s12889-021-10887-6

**Published:** 2021-05-06

**Authors:** Xian-zhi Fu, Qi-wei Sun, Chang-qing Sun, Fei Xu, Jun-jian He

**Affiliations:** 1 School of Political Science and Public Administration, Wuhan University, Wuhan, 430072 Hubei China; 2 School of International Education, Wuhan University, Wuhan, 430072 Hubei China; 3 Department of Social Medicine and Health Management, College of Public Health, Zhengzhou University, Zhengzhou, 450001 Henan China

**Keywords:** Catastrophic health expenditure, Fairlie nonlinear decomposition, Blinder-Oaxaca decomposition, China

## Abstract

**Background:**

The prevalence of chronic non-communicable diseases (NCDs) challenges the Chinese health system reform. Little is known for the differences in catastrophic health expenditure (CHE) between urban and rural households with NCD patients. This study aims to measure the differences above and quantify the contribution of each variable in explaining the urban-rural differences.

**Methods:**

Unbalanced panel data were obtained from the China Family Panel Studies (CFPS) conducted between 2012 and 2018. The techniques of Fairlie nonlinear decomposition and Blinder-Oaxaca decomposition were employed to measure the contribution of each independent variable to the urban-rural differences.

**Results:**

The CHE incidence and intensity of households with NCD patients were significantly higher in rural areas than in urban areas.

The urban-rural differences in CHE incidence increased from 8.07% in 2012 to 8.18% in 2018, while the urban-rural differences in CHE intensity decreased from 2.15% in 2012 to 2.05% in 2018. From 2012 to 2018, the disparity explained by household income and self-assessed health status of household head increased to some extent. During the same period, the contribution of education attainment to the urban-rural differences in CHE incidence decreased, while the contribution of education attainment to the urban-rural differences in CHE intensity increased slightly.

**Conclusions:**

Compared with urban households with NCD patients, rural households with NCD patients had higher risk of incurring CHE and heavier economic burden of diseases. There was no substantial change in urban-rural inequality in the incidence and intensity of CHE in 2018 compared to 2012. Policy interventions should give priority to improving the household income, education attainment and health awareness of rural patients with NCDs.

**Supplementary Information:**

The online version contains supplementary material available at 10.1186/s12889-021-10887-6.

## Background

Achieving universal health coverage, defined as ensuring that all people have access to essential health services without suffering financial constraints by 2030, is one of the key targets of the sustainable development goals (SDGs) [1, 2]. However, a global monitoring report released by the World Health Organization and World Bank reflects the situation of “poverty caused by illness” in the global population in 2017: (1) more than 122 million people were classified as “poor” (living on less than $3.10 a day) due to health care expenditure; (2) about 100 million people were pushed into “extremely poor” (living on less than $1.90 a day) because they have to pay for health care [3]. With the prevalence of chronic non-communicable diseases (NCDs) accompanied by accelerated population aging, increasing number of individuals worldwide will suffer from catastrophic health expenditure (CHE) in the future.

As the global epicenter of NCDs epidemic, China is under great pressure. A 2005 study estimated that NCDs had become the leading cause of death and disease burden in China, accounting for 80% of deaths and 70% of disability-adjusted life-years lost [4]. In 2015, NCDs contributed to 86.6% of all deaths and 70% of the total disease burden in China [5]. The heavy burden of NCDs has greatly increased the economic risks for many vulnerable groups in China.

The fundamental functions of a health system is not only to promote access to essential health care services, but also to improve the ability of households to withstand the financial catastrophe associated with illness [6]. The Chinese health system has been working to protect vulnerable households against CHE. In 2009, China’s new round of health system reform involved a series of policy measures, including the reduction of out-of-pocket (OOP) medical expenditure and expansion of basic health care coverage by 2020 [7, 8]. Three types of basic medical insurance schemes, including the Urban Employee Basic Medical Insurance (UEBMI), Urban Residents Basic Medical Insurance (URBMI) and New Rural Cooperative Medical Scheme (NRCMS), have been established to decrease the financial burden of NCDs on households. In 2013, more than 95% of residents were covered by basic medical insurance in China, which was a sign of universal coverage of basic medical insurance [9, 10]. In addition, supplementary medical insurance (SMI), including commercial medical insurance, public servant medical subsidy, enterprise supplementary medical subsidy, employee medical subsidy for large medical expenses, and employee mutual medical insurance, was established to meet the needs of residents for multiple levels of health services [11]. However, there was still evidence that medical expenditure due to NCDs played an important role in the main causes of poverty among rural households in China [12]. As NCDs are characterized by long treatment duration and high treatment costs [13], substantial financial hardships create obstacles to health services utilization for rural households with NCD patients in China, leading to further escalation of health problems. Therefore, it is necessary and urgent to pay attention to the CHE among rural households with NCD patients.

Several researches have investigated the financial catastrophe among individuals or households suffering from NCDs around the world. Three existing studies emphasized that households with NCD patients were in the high risk to incur CHE in China, Korea and Iran [9, 14, 15]. Gwatidzo (2017) found that adults aged 50 or above in India were less likely to incur CHE due to diabetes mellitus medication use compared to China [16]. Zhao (2019) identified that the CHE incidence among rural households with NCD patients notably exceeded the average level of urban households with NCD patients in China [17]. Xie (2017) verified the main reasons why households with members suffering from NCDs in rural China were prone to CHE [18]. To sum up, most of the studies have explored the CHE of households with NCD patients in rural areas of a country or in a whole country. However, there are seldom researches on the urban-rural differences in CHE among households with NCD patients and its influencing factors. In addition, understanding the urban-rural differences in the financial risks of NCD medical expenses and the factors related to the differences can prompt more effective efforts to reduce the economic risk of rural households with NCD patients.

The objectives of this study were as follows: (1) to measure the extent of CHE for urban and rural households with NCD patients, (2) to examine the urban-rural differences in the degree of CHE between the two groups, and (3) to quantify the contribution of each variable to the urban-rural differences.

## Methods

### Data source

This study was based on a publicly available database, the China Family Panel Studies (CFPS), which was conducted by the Institute of Social Science Survey (ISSS) of Peking University every 2 y from 2010 to 2018. The CFPS used a three-stage, stratified, probability-proportional-to-scale (PPS) random sampling method to select sample from 25 provinces in China. It was representative that the sample of CFPS representing 94.5% of the population in mainland China [19]. The questionnaire for CFPS involved a wide range of variables, such as demography characteristics, socioeconomic status, health status, health services utilization, family relationships and medical insurance and so on.

**Fig. 1:**
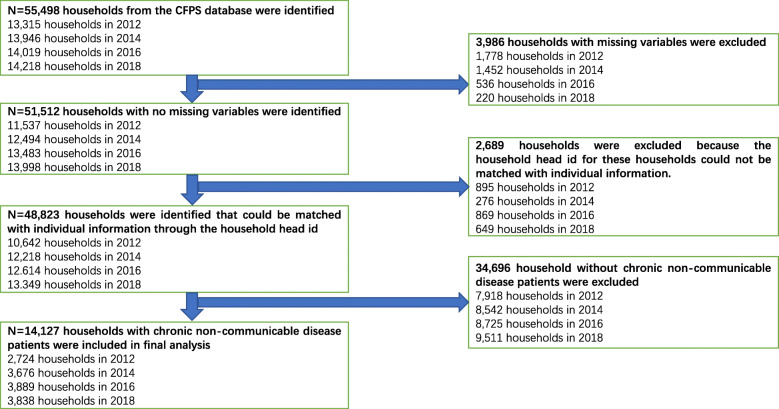
Flow chart of sample selection

We used four waves of data from the CFPS, which involved 13,315 households in 2012, 13,946 households in 2014, 14,019 households in 2016, and 14,218 households in 2018, respectively. The inclusion criteria for the interviewed households were as follows: (1) no missing variables; and (2) having members with NCDs (e.g., hypertension, diabetes, chronic lung disease, cancer or malignant tumor, liver disease, heart attack, stomach or other digestive disease, emotional nervousness or psychiatric problems, asthma, arthritis or rheumatism, and kidney disease). In this survey, NCDs were determined by whether a respondent had been diagnosed by a doctor within the previous 6 months? Family members were defined as those members who eat together in the household. Finally, 2724 households with NCD patients in 2012, 3676 households with NCD patients in 2014, 3889 households with NCD patients in 2016, and 3838 households with NCD patients in 2018 were specialized in this study, including 1224 households in urban areas and 1500 households in rural areas in 2012, 1782 households in urban areas and 1894 households in rural areas in 2014, 1847 households in urban areas and 2042 households in rural areas in 2016, and 1826 households in urban areas and 2012 households in rural areas in 2018. The detailed sampling process is shown in Fig. 1.

### Measurement of CHE

We referred to the studies of Wagstaff and van Doorslaer to determine the relevant indicators of measuring CHE [20, 21]. OOP medical expenditure only included direct medical expenditure made by any household members, and excluded indirect expenditure related to seeking health services (e.g., transportation, food, accommodation, lost productivity due to illness). Since the substitution of non-food household expenditure for total household expenditure partly avoided the measurement deviations that were often overlooked in poor households, we used non-food household expenditure as the denominator to calculate CHE [22, 23]. The non-food expenditure of a household is defined as the portion of total household expenditure excluding household food expenditure. According to exiting literature [17, 22, 24, 25], the threshold for CHE was defined as 40%. More specifically, if OOP medical expenditure of a household exceeded 40% of its non-food household expenditure, the household was classified as incurring CHE. A binary variable was defined to determine whether a household experienced CHE or not, as shown in formula (1):

1 $${E}_i=\left\{\begin{array}{c}0\kern0.5em if\kern0.5em \frac{T_i}{\left({x}_i-{f}_i\right)}< threshold\\ {}1\kern0.5em if\kern0.5em \frac{T_i}{\left({x}_i-{f}_i\right)}\ge threshold\end{array}\right.$$

where *T*_*i*_ means the OOP medical expenditure of household *i*, *x*_*i*_ is the total expenditure of household *i*, *f*_*i*_ stands for the food expenditure of household *i*, and threshold is defined as 40%. The calculation of CHE incidence and intensity can be specified as below:

2 $$H=\frac{1}{N}\sum \limits_{i=1}^N{E}_i$$

3 $$O=\frac{1}{N}\sum \limits_{i=1}^N{E}_i\left(\frac{T_i}{\left({x}_i-{f}_i\right)}-z\right)=\frac{1}{N}\sum \limits_{i=1}^N{O}_i$$

4 $$MPO=\frac{O}{H}\kern1.75em$$

where *N* represents the total sample size, *H* means the CHE incidence in the overall sample. CHE intensity is estimated by overshoot and mean positive overshoot (MPO). *O* stands for overshoot, which is the average percentage of OOP medical expenditure that exceeds a given threshold in the overall sample [26]. MPO indicates the average percentage of OOP medical expenditure in excess of the threshold among households incurring CHE [20]. The higher values of overshoot and MPO both stand for heavier financial burden of diseases for the household.

### Definitions of independent variables

Referring to the previous reports, we included the characteristics of each household and its household head into the regression model as independent variables [22, 23, 27–29]. Households characteristics involved six variables: the annual household income per capita, household size, receiving inpatient services, having members below 5 years old, having elderly members and geographic location. The characteristics of household head involved six variables: gender, education, marriage, self-assessed health status, basic medical insurance and SMI. We used the natural logarithm of the annual household income per capita to measure economic status of a household. All income and expenditure variables from 2014 to 2018 were deflated to 2012 using the corresponding consumer price index. In addition, there were only two forms of SMI in this study: (1) the form of commercial medical insurance operated and managed by commercial companies, and (2) the form in which industry organizations raise and manage their own funds in according with the principles of insurance. Table 1 presents the detailed descriptions of the above independent variables.

**Table 1:** Description of variables

Variables	Description
Household expenditure (CNT)	Total consumption expenditure of a household.
OOP medical expenditure (CNY)	Total out-of-pocket medical expenditure of a household.
Food expenditure (CNY)	Total food consumption expenditure of a household.
Household income (CNY)	The annual household income per capita.
Household size	The number of household members.
Inpatient	At least one household member received inpatient services in last year; Yes = 1; No = 0^a^.
Household members aged<=5	At least one household member below 5 years old; Yes = 1; No = 0^a^.
Household members aged> = 60	At least one household member over 60 years old; Yes = 1; No = 0^a^.
Geographic location	East = 1^a^; Central = 2; West = 3.
Gender of household head	Female = 0^a^; Male = 1.
Education of household head	Illiterate = 1^a^; Primary school = 2; Middle school = 3; High school and above = 4.
Marriage of household head	Married = 1; Unmarried = 0^a^.
Self-assessed health status of household head	Unhealthy = 0^a^; Healthy = 1.
Basic medical insurance	No medical insurance = 1^a^; UEBMI = 2; URBMI = 3; NRCMS = 4; Two kind of medical insurance = 5
SMI	The household head is covered by SMI; Yes = 1; No = 0^a^.

Note: ^a^ Reference group; *OOP* Out-of-pocket; *UEBMI* Urban Employee Basic Medical Insurance, *URBMI* Urban Residents Basic Medical Insurance, *NRCMS* New Rural Cooperative Medical Scheme, *SMI* Supplementary medical insurance

### Methodology

The Blinder-Oaxaca decomposition technique, proposed by Blinder and Oaxaca [30, 31], was applied in this study to analyze the contribution of each independent variable to the urban-rural differences in CHE. The implementation of decomposition analysis needs to be based on the relationship between CHE and a series of independent variables.

As CHE incidence (*E*_*i*_) is a binary variable, probit model is applied to estimate the effect of the independent variables on the CHE incidence. The specific regression model is shown below:

5 $${Y}^{\gamma }=F\left({X}^{\gamma }{\beta}^{\gamma}\right)$$

where *F* represents the cumulative distribution function of the standard normal distribution, superscript *γ* represents the rural or urban households, *Y* is the CHE incidence, *X* stands for the independent variables, and *β* denotes the regression coefficient.

Fairlie extended the technique of Blinder-Oaxaca decomposition to the application of nonlinear model [32, 33]. Given the probit regression model is a nonlinear regression model, this study employed the method of Fairlie nonlinear decomposition to decompose the urban-rural differences in CHE incidence between two groups into two components:

6 $${\overline{Y}}^R-{\overline{Y}}^U=\underset{Explained\ part}{\underbrace{\left[\sum \limits_{i=1}^{N^R}\frac{F\left({X}_i^R{\beta}^R\right)}{N^R}-\sum \limits_{i=1}^{N^U}\frac{F\left({X}_i^U{\beta}^R\right)}{N^U}\right]}}+\underset{Unexplained\ part}{\underbrace{\left[\sum \limits_{i=1}^{N^U}\frac{F\left({X}_i^U{\beta}^R\right)}{N^U}-\sum \limits_{i=1}^{N^U}\frac{F\left({X}_i^U{\beta}^U\right)}{N^U}\right]}}$$

Where superscript R represents the rural households, superscript U means the urban households. $\overline{Y}$ does not necessarily equal $F\left(\overline{X}\beta \right)$. The first term in formula (6) stands for the explained part of the urban-rural differences between two grousps, which is caused by the disparity in distribution of independent variables, and the second term represents the unexplained part due to the disparity in regression coefficient [34].

The detailed decomposition involves a natural one-to-one matching of cases between the two groups to identify the contribution of independent variables. The subsample was drawn from the majority group (rural households), and matched the minority group (urban households) based on the ranking of CHE incidence. The contribution of variable *X*_1_ to the urban-rural differences in CHE incidence is estimated as follows:

7 $$\frac{1}{N^U}\sum \limits_{i=1}^{N^U}F\left({\alpha}^{\ast }+{X}_{1i}^R{\beta}_1^{\ast }+{X}_{2i}^R{\beta}_2^{\ast}\right)-F\left({\alpha}^{\ast }+{X}_{1i}^U{\beta}_1^{\ast }+{X}_{2i}^R{\beta}_2^{\ast}\right)\kern0.75em$$

Where *β*^∗^ stands for the regression coefficient from the probit model for the overall sample. It should be noted that the results are sensitive to the order of independent variables in the decomposition of nonlinear model [34]. Following Fairlie [33], independent variables were randomly ordered in the decomposition of nonlinear model. This study repeated the above steps 1000 times to obtain the average value of decomposition results, representing the contribution of each independent variable.

Similarly, the contribution of *X*_2_ to the urban-rural differences in CHE incidence is calculated as follows:

8 $$\frac{1}{N^U}\sum \limits_{i=1}^{N^U}F\left({\alpha}^{\ast }+{X}_{1i}^U{\beta}_1^{\ast }+{X}_{2i}^R{\beta}_2^{\ast}\right)-F\left({\alpha}^{\ast }+{X}_{1i}^U{\beta}_1^{\ast }+{X}_{2i}^U{\beta}_2^{\ast}\right)$$

In addition, since the CHE intensity (*O*_*i*_) is a continuous variable, multiple linear regression is used to analyze the factors affecting the CHE intensity. The specific regression model can be written as:

9 $${Y}^{\gamma }={X}^{\gamma }{\beta}^{\gamma }+{\varepsilon}^{\gamma }$$

where *Y* represents the CHE intensity, *X* stands for a vector of independent variables, *β* is a vector of regression coefficient including intercept, and ε denotes the random error term.

The contribution of each independent variable to the urban-rural differences in CHE intensity was divided into two components using two-fold Blinder-Oaxaca decomposition approach [35, 36]:

10 $${\overline{Y}}^R-{\overline{Y}}^U=\underset{Explained\ part}{\underbrace{\left({\overline{X}}^R-{\overline{X}}^U\right){\beta}^{\ast }}}+\underset{Unexplained\ part}{\underbrace{\left[{\overline{X}}^R\left({\beta}^R-{\beta}^{\ast}\right)+{\overline{X}}^U\left({\beta}^{\ast }-{\beta}^U\right)\right]}}$$

Where *β*^∗^ denotes the regression coefficient from the multiple linear regression for the overall sample, $\overline{X}$ represents the corresponding covariate means of the independent variables. The first term indicates the explained part, representing the contribution attributable to group disparity in distribution of independent variables, and the second term indicates the unexplained part, representing the contribution attributable to group disparity in regression coefficient.

It is necessary to emphasize that the Fairlie nonlinear decomposition and Blinder-Oaxaca decomposition are mainly applied to analyze cross-sectional data. Therefore, the regression coefficients needed to calculate the decomposition results were mainly derived from the cross-sectional analysis of the corresponding years. However, considering the superiority of the panel regression model for causal inference and the limited length of this paper, we only presented the analysis results of the panel regression model. In general, panel regression model can be categorized as fixed effects model and random effects model. Fixed effects model would be a poor choice in a situation where independent variables don’t change much over time [11]. In this study, most of the interviewed households included variables (e.g., geographic location, gender of the household head, etc.) that did not change over time. Given the strict samples inclusion criteria for the fixed effects model, we applied random effects panel model for regression analysis.

All statistical analyses were performed in STATA software version 15.1, and *p* < 0.05 was considered statistically significant.

## Results

### Descriptive statistics

Table 2 shows the summary statistics for general characteristics of the urban and rural households with NCD patients. From 2012 to 2018, the mean household size in rural areas was greater than that in urban areas. Meanwhile, the rural households had higher probability in receiving inpatient services in the last 12 months, having children below 5 years old, and having elderly members. In terms of the coverage of basic medical insurance, the proportion of household head with UEBMI and URBMI was higher in urban areas than in rural areas, while the proportion of household head with NRCMS was higher in rural areas than in urban areas. With respect to the coverage of SMI, the proportion of household head having SMI was higher in urban areas in comparison with the rural areas. The percentage of households having female household head was higher in urban areas than in rural areas. In urban areas, the highest percentage of households were located in the east, while in rural areas, the highest percentage of households were located in the west. The education level of household heads in urban areas was mainly middle school or high school and above, while the highest proportion of household head in rural areas was illiterate.

**Table 2:** Summary statistics of variables

Variables	2012				2014			
	Urban areas		Rural areas		Urban areas		Rural areas	
	Mean(*N*)	S.D. (%)	Mean(*N*)	S.D. (%)	Mean(*N*)	S.D. (%)	Mean(*N*)	S.D. (%)
Household expenditure (CNY)	50,926.38	76,144.12	32,051.80	34,346.17	58,323.30	85,216.30	37,469.02	51,815.58
OOP medical expenditure (CNY)	6056.99	16,110.78	4895.33	11,161.51	6749.66	15,948.90	6043.14	12,331.56
Food household expenditure (CNY)	17,327.07	15,105.43	12,960.50	11,634.00	20,079.08	16,056.22	10,619.55	11,161.20
Household size	3.68	1.67	4.29	1.92	3.62	1.72	4.20	1.92
Inpatient								
Yes	450	36.76	554	36.93	737	41.36	825	43.56
No^1^	774	63.24	946	63.07	1045	58.64	1069	56.44
Household members aged<=5								
Yes	205	16.75	357	23.80	290	16.27	427	22.54
No^1^	1019	83.25	1143	76.20	1492	83.73	1467	77.46
Household members aged> = 60								
Yes	583	47.63	762	50.80	983	55.16	1057	55.81
No^1^	641	52.37	738	49.20	799	44.84	837	44.19
Geographic location								
East^1^	629	51.39	509	33.93	852	47.81	664	35.06
Central	401	32.76	451	30.07	595	33.39	578	30.52
West	194	15.85	540	36.00	335	18.80	652	34.42
Gender of household head								
Female^1^	648	52.94	582	38.80	932	52.30	785	41.45
Male	576	47.06	918	61.20	850	47.70	1109	58.55
Education of household head								
Illiterate^1^	256	20.92	585	39.00	327	18.35	657	34.69
Primary school	225	18.38	410	27.33	368	20.65	572	30.20
Middle school	367	29.98	364	24.27	549	30.81	481	25.40
High school and above	376	30.72	141	9.40	538	30.19	184	9.71
Marriage of household head								
Married	1094	89.38	1370	91.33	1549	86.92	1710	90.29
Unmarried^1^	130	10.62	130	8.67	233	13.08	184	9.71
Self-assessed health status of household head								
Unhealthy^1^	685	55.96	821	54.73	866	48.60	938	49.52
Healthy	539	44.04	679	45.27	916	51.40	956	50.48
Basic medical insurance								
No medical insurance^1^	175	14.30	98	6.53	150	8.42	95	5.02
UEBMI	382	31.21	64	4.27	570	31.99	82	4.33
URBMI	226	18.46	36	2.40	316	17.73	53	2.80
NRCMS	429	35.05	1297	86.47	722	40.52	1657	87.49
Two kinds of medical insurance	12	0.98	5	0.33	24	1.35	7	0.37
SMI								
Yes	11	0.90	3	0.20	38	2.13	15	0.79
No^1^	1213	99.10	1497	99.80	1744	97.87	1879	99.21
Observations	1224	100	1500	100	1782	100	1894	100
Variables	2016				2018			
	Urban areas		Rural areas		Urban areas		Rural areas	
	Mean(N)	S.D. (%)	Mean(N)	S.D. (%)	Mean(N)	S.D. (%)	Mean(N)	S.D. (%)
Household expenditure (CNY)	67,405.13	120,825.70	40,183.21	47,160.57	71,220.84	77,596.94	38,913.09	40,913.34
OOP medical expenditure (CNY)	8329.21	20,640.78	6760.40	14,725.37	7832.77	17,389.48	6746.01	15,173.89
Food household expenditure (CNY)	21,125.59	16,339.34	10,601.42	11,455.37	22,927.93	18,662.01	10,933.08	10,910.03
Household size	3.72	1.84	4.27	2.02	3.71	1.91	4.12	2.06
Inpatient								
Yes	833	45.10	958	46.91	842	46.11	989	49.16
No^1^	1014	54.90	1084	53.09	984	53.89	1023	50.84
Household members aged<=5								
Yes	295	15.97	440	21.55	309	16.92	378	18.79
No^1^	1552	84.03	1602	78.45	1517	83.08	1634	81.21
Household members aged> = 60								
Yes	1096	59.34	1215	59.50	1107	60.62	1291	64.17
No^1^	751	40.66	827	40.50	719	39.38	721	35.83
Geographic location								
East^1^	811	43.91	626	30.66	903	49.45	629	31.26
Central	660	35.73	653	31.98	540	29.57	608	30.22
West	376	20.36	763	37.37	383	20.97	775	38.52
Gender of household head								
Female^1^	984	53.28	905	44.32	927	50.77	871	43.29
Male	863	46.72	1137	55.68	899	49.23	1141	56.71
Education of household head								
Illiterate^1^	394	21.33	754	36.92	312	17.09	699	34.74
Primary school	380	20.57	584	28.60	353	19.33	555	27.58
Middle school	537	29.07	492	24.09	589	32.26	520	25.84
High school and above	536	29.02	212	10.38	572	31.33	238	11.83
Marriage of household head								
Married	1593	86.25	1798	88.05	1580	86.53	1736	86.28
Unmarried^1^	254	13.75	244	11.95	246	13.47	276	13.72
Self-assessed health status of household head								
Unhealthy^1^	951	51.49	1132	55.44	855	46.82	1009	50.15
Healthy	896	48.51	910	44.56	971	53.18	1003	49.85
Basic medical insurance								
No medical insurance	164	8.88	92	4.51	154	8.43	120	5.96
UEBMI	522	28.26	70	3.43	534	29.24	71	3.53
URBMI	337	18.25	35	1.71	351	19.22	41	2.04
NRCMS	806	43.64	1838	90.01	770	42.17	1774	88.17
Two kinds of medical insurance	18	0.97	7	0.34	17	0.93	6	0.30
SMI								
Yes	28	1.52	11	0.54	33	1.81	18	0.89
No^1^	1819	98.48	2031	99.46	1793	98.19	1994	99.11
Observations	1847	100	2042	100	1826	100	2012	100

Note: ^1^ Reference group; *OOP* Out-of-pocket; *UEBMI* Urban Employee Basic Medical Insurance; *URBMI* Urban Residents Basic Medical Insurance, *NRCMS* New Rural Cooperative Medical Scheme, *SMI* Supplementary medical insurance

### CHE incidence and intensity

Table 3 illustrates CHE incidence and intensity of urban and rural households with NCD patients. In 2018, 17.96% of households in urban areas experienced CHE. Meanwhile, the overshoot of urban households was 3.98% in 2018, suggesting that the average percentage of OOP medical expenditure that exceeded the given threshold over all urban households was 3.98%. The MPO for urban households was 22.16% in 2018, meaning that if the burden of overshoot was divided equally by all urban households incurring CHE, the average extent of exceeding given threshold was 22.16%. Each of the other row could be interpreted in a similar pattern for rural/urban households with NCD patients in 2012/2014/2016/2018.

**Table 3:** CHE incidence and intensity in rural and urban households with NCD patients

		Urban areas	Rural areas
2012	Incidence	19.53	27.60
	Overshoot	4.24	6.40
	MPO	21.71	23.19
2014	Incidence	18.80	26.03
	Overshoot	4.27	5.75
	MPO	22.71	22.09
2016	Incidence	20.19	26.00
	Overshoot	4.44	6.21
	MPO	21.99	23.88
2018	Incidence	17.96	26.14
	Overshoot	3.98	6.03
	MPO	22.16	23.07

Note: *CHE* Catastrophic health expenditure, *MPO* Mean positive overshoot

From 2012 to 2018, the CHE incidence decreased from 19.53 to 17.96% in urban households with NCD patients and decreased from 27.60 to 26.14% in rural households with NCD patients. The overshoot decreased from 4.24 to 3.98% in urban households with NCD patients and decreased from 6.40 to 6.03% in rural households with NCD patients. The MPO increased from 21.71 to 22.16% in urban households with NCD patients and decreased from 23.19 to 23.07% in rural households with NCD patients. None of the indicators showed a steady upward or downward trend.

### Associated factors of CHE incidence

Table 4 presents the results of random effects panel probit regression model for factors associated with CHE incidence in urban and rural households with NCD patients. Household income and household size were negatively associated with CHE incidence. Better self-rated health status and higher education attainment of household head significantly decreased the CHE incidence, while receiving inpatient services in the last 12 months and having elderly members significantly increased the occurrence of exposure to CHE. The geographic location of west was negatively correlated with CHE incidence. Having children below 5 years old significantly increased the CHE incidence of rural households. SMI was negatively associated with the CHE incidence of urban households. Meanwhile, UEBMI and URBMI were negatively associated with CHE incidence, while NRCMS was positively correlated with CHE incidence. However, none of the three types of basic medical insurance had a significant effect on the CHE incidence.

**Table 4:** Association between factors and CHE incidence in rural and urban households with NCD patients

Variables	Urban areas		Rural areas	
	dy/dx	Std. Err.	dy/dx	Std. Err.
Household income	−0.0328**	0.0044	−0.0281**	0.0044
Household size	−0.0362**	0.0032	−0.0457**	0.0030
Inpatient, yes	0.1720**	0.0087	0.1871**	0.0091
Household members aged<=5, yes	−0.0093	0.0153	0.0330*	0.0141
Household members aged> = 60, yes	0.0604**	0.0099	0.0778**	0.0107
Geographic location				
East^1^				
Central	−0.0059	0.0107	−0.0225	0.0128
West	−0.0285*	0.0130	−0.0699**	0.0127
Gender of household head, male	−0.0018	0.0092	0.0095	0.0102
Education of household head				
Illiterate^1^				
Primary school	−0.0359**	0.0131	−0.0493**	0.0124
Middle school	−0.0799**	0.0128	−0.0673**	0.0137
High school and above	− 0.1035**	0.0144	− 0.0596**	0.0196
Marriage of household head, married	−0.0150	0.0131	0.0069	0.0155
Self-assessed health status of household head, healthy	−0.0804**	0.0091	−0.0724**	0.0098
Basic medical insurance				
No medical insurance^1^				
UEBMI	−0.0160	0.0170	−0.0074	0.0342
URBMI	−0.0059	0.0176	−0.0280	0.0395
NRCMS	0.0067	0.0161	0.0328	0.0214
Two kinds of medical insurance	−0.1214	0.0626	−0.0966	0.1068
SMI, yes	−0.0889*	0.0451	− 0.0918	0.0684

Note: ^1^ Reference group; *UEBMI* Urban Employee Basic Medical Insurance; *URBMI* Urban Residents Basic Medical Insurance; *NRCMS* New Rural Cooperative Medical Scheme; *SMI* Supplementary medical insurance; The dy/dx in brackets indicates the marginal effect; * *p* < 0.05; ** *p* < 0.01

### Associated factors of CHE intensity

The associated factors of the CHE intensity (*O*_*i*_) are shown in Table 5. These results indicated a significant negative association between CHE intensity and household income, and between CHE intensity and household size. Better self-rated health status and higher education attainment of household head significantly decreased the CHE intensity, while receiving inpatient services in the last 12 months and having elderly members significantly increased the CHE intensity. The geographic location of west significantly decreased the CHE intensity. SMI was negatively associated with the CHE intensity of rural households. Meanwhile, URBMI was negatively correlated with CHE intensity, while NRCMS was positively associated with CHE intensity. UEBMI was negatively correlated with CHE intensity of urban households, and was positively associated with CHE intensity of rural households. However, none of the three types of basic medical insurance had a significant effect on the CHE intensity.

**Table 5:** Association between factors and CHE intensity in rural and urban households with NCD patients

Variables	Urban areas		Rural areas	
	dy/dx	Std. Err.	dy/dx	Std. Err.
Household income	−0.0098**	0.0014	−0.0105**	0.0017
Household size	−0.0104**	0.0009	−0.0105**	0.0012
Inpatient, yes	0.0490**	0.0027	0.0538**	0.0034
Household members aged<=5, yes	−0.0023	0.0040	0.0001	0.0050
Household members aged> = 60, yes	0.0153**	0.0028	0.0206**	0.0042
Geographic location				
East^1^				
Central	−0.0057	0.0032	−0.0107	0.0060
West	−0.0127**	0.0038	−0.0245**	0.0058
Gender of household head, male	0.0047	0.0027	0.0032	0.0038
Education of household head				
Illiterate^1^				
Primary school	−0.0191**	00043	− 0.0152**	0.0051
Middle school	−0.0275**	0.0041	−0.0197**	0.0055
High school and above	−0.0304**	0.0044	−0.0106	0.0076
Marriage of household head, married	−0.0018	0.0041	0.0037	0.0063
Self-assessed health status of household head, healthy	−0.0203**	0.0027	−0.0159**	0.0036
Basic medical insurance				
No medical insurance^1^				
UEBMI	−0.0082	0.0049	0.0025	0.0119
URBMI	−0.0034	0.0052	−0.0076	0.0141
NRCMS	0.0033	0.0048	0.0134	0.0074
Two kinds of medical insurance	−0.0228	0.0130	0.0216	0.0299
SMI, yes	−0.0137	0.0102	−0.0405*	0.0206

Note: ^1^ Reference group; *UEBMI* Urban Employee Basic Medical Insurance; *URBMI* Urban Residents Basic Medical Insurance; *NRCMS* New Rural Cooperative Medical Scheme; *SMI* Supplementary medical insurance; The dy/dx in brackets indicates the marginal effect; * *p* < 0.05; ** *p* < 0.01

### Aggregate decomposition

Table 6 displays the results for aggregate decomposition of the urban-rural differences in CHE incidence and intensity (*O*_*i*_) among households with NCD patients. The explained disparity of CHE incidence increased from 14.87% in 2012 to 57.95% in 2018, a relative increase of 289.71%. The corresponding values of CHE intensity rose from 59.53% in 2012 to 88.29% in 2018, a relative increase of 48.31%. None of the indicators showed a steady upward or downward trend.

**Table 6:** Aggregate decomposition of the urban-rural differences in CHE incidence and intensity among households with NCD patients

		Total differences		Explained part		Unexplained part	
		Coefficient	Percent	Coefficient	Percent	Coefficient	Percent
2012	CHE incidence	0.0807	100.00	0.0120	14.87	0.0687	85.13
	CHE intensity	0.0215	100.00	0.0128	59.53	0.0087	40.47
2014	CHE incidence	0.0723	100.00	0.0411	56.85	0.0312	43.15
	CHE intensity	0.0148	100.00	0.0067	45.27	0.0081	54.73
2016	CHE incidence	0.0581	100.00	0.0231	39.76	0.0350	60.24
	CHE intensity	0.0178	100.00	0.0114	64.04	0.0064	35.96
2018	CHE incidence	0.0818	100.00	0.0474	57.95	0.0344	42.05
	CHE intensity	0.0205	100.00	0.0181	88.29	0.0024	11.71

Note: *CHE* Catastrophic health expenditure

### Decomposition of contribution of all explanatory variables

The urban-rural differences in CHE incidence and intensity (*O*_*i*_) among households with NCD patients is further decomposed into the contribution of each variable, as shown in Tables 7 and 8.

**Table 7:** Detailed decomposition of the urban-rural differences in CHE incidence among households with NCD patients

Variables	2012		2014		2016		2018	
	Explained part	Percent (%)	Explained part	Percent (%)	Explained part	Percent (%)	Explained part	Percent (%)
Household income	0.0066	8.18	0.0176**	24.34	0.0193**	33.22	0.0445**	54.40
Household size	−0.0242**	−29.99	− 0.0177**	−24.48	− 0.0208**	−35.80	− 0.0135**	−16.50
Inpatient, yes	− 0.0032	−3.97	0.0040*	5.53	0.0020	3.44	0.0034	4.16
Household members aged<=5, yes	0.0008	0.99	0.0009	1.24	0.0019	3.27	0.0005	0.61
Household members aged> = 60, yes	0.0020	2.48	−0.0003	− 0.41	0.0000	0.00	0.0029**	3.55
Geographic location								
East^1^								
Central	−0.0003	− 0.37	0.0011	1.52	0.0016	2.75	0.0001	0.12
West	−0.0122*	−15.12	−0.0089**	− 12.31	− 0.0110**	− 18.93	−0.0082*	− 10.02
Gender of household head, male	0.0005	0.62	0.0005	0.69	0.0025	4.30	−0.0005	−0.61
Education of household head								
Illiterate^1^								
Primary school	−0.0045*	−5.58	−0.0052**	−7.19	− 0.0024	−4.13	− 0.0023	−2.81
Middle school	0.0052*	6.44	0.0067**	9.27	0.0040*	6.88	0.0032	3.91
High school and above	0.0197*	24.41	0.0117	16.18	0.0074	12.74	0.0030	3.67
Marriage of household head, married	−0.0012	−1.49	0.0007	0.97	0.0004	0.69	0.0000	0.00
Self-assessed health status of household head, healthy	−0.0008	− 0.99	0.0010	1.38	0.0031**	5.34	0.0027**	3.30
Basic medical insurance								
No medical insurance^1^								
UEBMI	−0.0179	−22.18	0.0177	24.48	− 0.0136	−23.41	0.0142	17.36
URBMI	−0.0029	−3.59	0.0133	18.40	0.0012	2.07	−0.0015	−1.83
NRCMS	0.0451	55.89	−0.0033	−4.56	0.0266	45.78	0.0000	0.00
Two kinds of medical insurance	–	–	–	–	−0.0001	−0.17	− 0.0007	−0.86
SMI, yes	−0.0007	−0.87	0.0013	1.80	0.0010	1.72	−0.0004	−0.49

Note: ^1^ Reference group; *UEBMI* Urban Employee Basic Medical Insurance, *URBMI* Urban Residents Basic Medical Insurance; *NRCMS* New Rural Cooperative Medical Scheme; *SMI* Supplementary medical insurance; * *p* < 0.05; ** *p* < 0.01

**Table 8:** Detailed decomposition of the urban-rural differences in CHE intensity among households with NCD patients

**Variables**	**2012**				**2014**			
	**Explained part**	**Percent (%)**	**Unexplained part**	**Percent (%)**	**Explained part**	**Percent (%)**	**Unexplained part**	**Percent (%)**
Household income	0.0067**	31.16	0.0183	85.12	0.0066**	44.59	0.0121	81.76
Household size	−0.0081**	−37.67	−0.0146	−67.91	− 0.0059**	− 39.86	−0.0116	−78.38
Inpatient, yes	0.0001	0.47	0.0042	19.53	0.0012	8.11	−0.0051	−34.46
Household members aged<=5, yes	−0.0006	−2.79	−0.0017	−7.91	− 0.0001	−0.68	0.0027	18.24
Household members aged> = 60, yes	0.0008	3.72	0.0045	20.93	0.0001	0.68	0.0033	22.30
Geographic location								
East^1^								
Central	−0.0001	−0.47	−0.0024	−11.16	0.0004	2.70	−0.0048	−32.43
West	−0.0045**	−20.93	−0.0048	−22.33	− 0.0044**	−29.73	−0.0009	−6.08
Gender of household head, male	0.0013*	6.05	−0.0026	−12.09	0.0005	3.38	0.0006	4.05
Education of household head								
Illiterate^1^								
Primary school	−0.0014*	−6.51	0.0020	9.30	−0.0016**	−10.81	0.0023	15.54
Middle school	0.0012*	5.58	0.0041	19.07	0.0016**	10.81	0.0034	22.97
High school and above	0.0035*	16.28	0.0006	2.79	0.0043**	29.05	0.0048	32.43
Marriage of household head, married	−0.0001	−0.47	−0.0136	−63.26	− 0.0003	−2.03	−0.0207	− 139.86
Self-assessed health status of household head, healthy	−0.0003	−1.40	0.0003	1.40	0.0002	1.35	0.0039	26.35
Basic medical insurance								
No medical insurance^1^								
UEBMI	0.0015	6.98	−0.0001	−0.47	0.0004	2.70	−0.0005	−3.38
URBMI	−0.0009	−4.19	−0.0005	−2.33	0.0001	0.68	−0.0011	−7.43
NRCMS	0.0136	63.26	−0.0062	−28.84	0.0031	20.95	0.0026	17.57
Two kinds of medical insurance	0.0002	0.93	−0.0001	− 0.47	0.0002	1.35	−0.0002	−1.35
SMI, yes	−0.0001	−0.47	0.0003	1.40	0.0003	2.03	−0.0001	−0.68
Constant			0.0210	97.67			0.0174	117.57
**Variables**	**2016**				**2018**			
	**Explained part**	**Percent (%)**	**Unexplained part**	**Percent (%)**	**Explained part**	**Percent (%)**	**Unexplained part**	**Percent (%)**
Household income	0.0085**	47.75	0.0227	127.53	0.0158**	77.07	−0.1813*	− 884.39
Household size	−0.0063**	−35.39	− 0.0043	−24.16	−0.0045**	−21.95	− 0.0002	−0.98
Inpatient, yes	0.0010	5.62	0.0056	31.46	0.0014	6.83	0.0056	27.32
Household members aged<=5, yes	0.0002	1.12	0.0006	3.37	0.0000	0.00	−0.0002	−0.98
Household members aged> = 60, yes	0.0000	0.00	0.0029	16.29	0.0008*	3.90	0.0051	24.88
Geographic location								
East^1^								
Central	0.0004	2.25	−0.0056	−31.46	0.0000	0.00	0.0057*	27.80
West	−0.0025**	−14.04	−0.0062*	−34.83	− 0.0023*	−11.22	0.0018	8.78
Gender of household head, male	0.0006	3.37	0.0016	8.99	−0.0004	−1.95	−0.0083	−40.49
Education of household head								
Illiterate^1^								
Primary school	−0.0015**	−8.43	0.0039	21.91	−0.0013*	−6.34	−0.0046	−22.44
Middle school	0.0010*	5.62	0.0014	7.87	0.0013*	6.34	−0.0004	−1.95
High school and above	0.0049**	27.53	0.0061*	34.27	0.0036**	17.56	0.0029	14.15
Marriage of household head, married	0.0000	0.00	0.0006	3.37	0.0000	0.00	0.0158	77.07
Self-assessed health status of household head, healthy	0.0008*	4.49	−0.0034	−19.10	0.0008*	3.90	−0.0030	−14.63
Basic medical insurance								
No medical insurance^1^								
UEBMI	0.0006	3.37	0.0035	19.66	0.0038	18.54	0.0034	16.59
URBMI	0.0009	5.06	0.0015	8.43	0.0012	5.85	0.0012	5.85
NRCMS	0.0024	13.48	0.0234*	131.46	−0.0019	−9.27	0.0060	29.27
Two kinds of medical insurance	0.0001	0.56	0.0004	2.25	−0.0001	−0.49	0.0006	2.93
SMI, yes	0.0003**	1.69	−0.0001	−0.56	− 0.0001	−0.49	0.0003	1.46
Constant			−0.0482	−270.79			0.1520	741.46

Note: ^1^ Reference group; *OOP* Out-of-pocket; *UEBMI* Urban Employee Basic Medical Insurance; *URBMI* Urban Residents Basic Medical Insurance, *NRCMS* New Rural Cooperative Medical Scheme, *SMI* Supplementary medical insurance; * *p* < 0.05; ** *p* < 0.01

With respect to the urban-rural differences in CHE incidence in 2012, the explained part was mainly attributed to household size (− 29.99%), geographic location (west, − 15.12%) and education of household head (primary school, − 5.58%; middle school, 6.44%; high school and above, 24.41%). The main contribution to the explained disparity in CHE incidence in 2018 was associated with household income (54.40%), household size (− 16.50%), having elderly members (3.55%), geographic location (west, − 10.02%), and self-rated health status of household head (3.30%).

With regard to the explained disparity of CHE intensity in 2012, the main contributors were household income (31.16%), household size (− 37.67%), geographic location (west, − 20.93%), gender of household head (6.05%), and education of household head (primary school, − 6.51%; middle school, 5.58%; high school and above, 16.28%). In 2018, the explained disparity in CHE intensity was mainly associated with household income (77.07%), household size (− 21.95%), having elderly members (3.90%), geographic location (west, − 11.22%), education of household head (primary school, − 6.34%; middle school, 6.34%; high school and above, 17.56%), and self-assessed health status of household head (3.90%).

## Discussion

By analyzing the national representative unbalanced panel data collected between 2012 and 2018 from the CFPS, this study estimates the extent of CHE for urban and rural households with NCD patients, as well as the differences in the degree of CHE between the two groups.

Here, we found that the CHE incidence of households with NCD patients in urban and rural areas were 17.96 and 26.14%, respectively, which are much higher than the results of another study on the overall proportion of households incurring CHE in China (urban households: 13.06%; rural households: 17.70%) [17]. It indicates that the risk tolerance of households with NCD patients to OOP medical expenditure is lower than the average level of Chinese households. Our results also showed that the households with NCD patients had higher incidence and intensity of CHE in rural areas than in urban areas, demonstrating that rural households with NCD patients have higher risk of incurring CHE and heavier economic burden of diseases.

Using regression analysis to examine the relevant influencing factors for CHE incidence and intensity from 2012 to 2018, this research identified several key determinants reported in prior studies (e.g., household income, household size, having children below 5 years old, having elderly members, education of household head, receiving inpatient services in the last 12 months) [10, 22, 23, 37]. Specifically, higher annual household income per capita, larger household size and higher education level of household head protected against CHE in urban and rural households with NCD patients. Conversely, households utilizing inpatient services, having elderly members and with poor self-assessed health status of household head had higher risk of incurring CHE and heavier economic burden of diseases. Having children below 5 years old may increase the likelihood of incurring CHE for rural household with NCD patients. Meanwhile, this study found that the geographic location of west reduced the risk of incurring CHE and financial burden of diseases in urban and rural households with NCD patients. One potential explanation is that households in western China are prone to forgo needed health services due to their low income [38].

None of the three types of basic medical insurance schemes, including UEBMI, URBMI and NRCMS, significantly reduced the incidence and intensity of CHE in both two groups, which is consistent with some existing literature [11, 22, 39–41]. The weak effect of basic medical insurance in reducing the incidence and intensity of CHE could be attributed to the relatively lower level of scope and actual reimbursement rate, as well as the heavy economic burden of NCDs [23]. The analysis of individual database showed that the OOP medical expenditure as a percentage of total medical expenditure was greater than 40% for both urban and rural patients with NCDs covered by basic medical insurance from 2014 to 2018 (Supplementary Table 1).

Meanwhile, we also found that the NRCMS provided a lower level of health benefits for patients with NCDs compared to the UEBMI and URBMI (Table 4, Table 5 and Supplementary Table 1). Given the special nature of NCDs, local governments in China had established a special outpatient reimbursement system to compensate the medical expenses of patients with critical NCDs. According to the funding levels of the different basic medical insurance, the types of diseases to be included in the list were identified, the corresponding reimbursement rates and ceiling levels were set, and patients with critical NCDs were compensated. The statistical results showed that the per capita funding level of the NRCMS in 2018 was 654.6 CNY, which is lower than URBMI (695.7 CNY) and UEBMI (4273.2 CNY) [42]. This was the main reason why the groups covered by NRCMS were in a relatively disadvantaged position. In order to solve the above problems, relevant suggestions are shown as follows: (1) to strengthen the government’s responsibility for basic medical insurance schemes, especially for the NRCMS, (2) to gradually include more critical NCDs into the list of diseases for outpatient critical illnesses, and (3) to integrate different medical insurance schemes to break through the barriers between different basic medical insurance schemes.

As the supplementary form of basic health insurance, SMI usually reimbursed patients for medical expenses in the form of “secondary compensation”. Our research found that SMI could reduce the incidence and intensity of CHE to some extent, but its effect was not particularly stable in terms of statistical significance. Given that SMI is characterized by voluntary participation, one plausible reason for this phenomenon is the low coverage rate of SMI [43, 44]. The coverage rate of SMI in urban households with NCD patients increased from 0.90% in 2012 to 1.81% in 2018, while the coverage rate of SMI in rural households with NCD patients increased from 0.20% in 2012 to 0.89% in 2018 (Table 2). Therefore, this study deems that the Chinese government should encourage the development of SMI to form a multi-dimensional medical insurance system to further alleviate the financial burden of illness for patients with NCDs.

From 2012 to 2018, the increase of the explained disparity offset the reduction of the unexplained disparity, resulting in a slight increase of the urban-rural differences in the CHE incidence. During the same period, the reduction of the unexplained disparity offset the increase of the explained disparity, resulting in a slight decrease of the urban-rural differences in the CHE intensity.

More importantly, this article identified major contributors to explain the urban-rural differences in CHE incidence and intensity among households with NCD patients. Specifically, household income made the largest positive contribution to the urban-rural differences. From 2012 to 2018, the disparity explained by household income gradually increased, which can be attributed to the increase in the income gap between urban and rural households with NCD patients. Similarly, the education attainment and self-assessed health status of household head also had positive contribution. From 2012 to 2018, the contribution of education attainment to the urban-rural differences in CHE incidence decreased, while the contribution of education attainment to the urban-rural differences in CHE intensity increased slightly. During the same period, the contribution of self-assessed health status to the urban-rural differences in CHE incidence and intensity increased slightly. From the perspective of policymakers, any intervention aimed at decreasing this disparity may be effective if they focus on the observable characteristics mentioned above. The specific suggestions are as follows: (1) poverty alleviation department should resolutely implement “targeted poverty alleviation” strategy to effectively improve the income level of rural households with NCDs; (2) education department should promote the construction of rural education to improve the education level of rural population; (3) propaganda department should strengthen the publicity of NCDs in rural areas to raise the health awareness of rural patients with NCDs.

In addition, the observed characteristics such as household size and geographic location of the west area had an opposite effect in explaining the urban-rural differences. From 2012 to 2018, the contribution of above characteristics to the reduction of the urban-rural differences declined to some extent. If the urban-rural disparity is further reduced in terms of above characteristics, the urban-rural differences in CHE incidence and intensity will be wilder.

The decomposition results regarding the various types of medical insurance schemes were not satisfactory. SMI made minor contribution to the increase of urban-rural differences in CHE incidence and intensity, and its effect was not particularly stable in terms of statistical significance. None of the three types of basic medical insurance had a significant effect on the urban-rural differences in CHE incidence and intensity.

The study is not without its limitations. First, various characteristics (e.g., the levels of medical institution, actual reimbursement rate of medical insurance, distance to the nearest medical institution) can significantly affect CHE in the reports of other scholars [22, 23, 45]. However, the absence of relevant indicators in the database or the inconsistency in the caliber of indicators between different years lead to some unexplained urban-rural differences in incidence and intensity of CHE. Second, the present research uses a conservative method to estimate the OOP medical expenditure, resulting in indirect expenditure (e.g., transportation, food, accommodation, lost productivity due to illness) not being included [10, 29]. Therefore, we underestimated the CHE incidence and intensity to a certain extent. Third, since this study involves self-reported information about health status of household head, the possibility of reporting errors cannot be ruled out.

## Conclusion

In conclusion, the present study suggested that rural households with NCD patients had higher CHE incidence and intensity than urban ones. None of the three types of basic medical insurance schemes significantly reduced the incidence and intensity of CHE in both two groups. In particular, NRCMS provided a lower level of health benefits for patients with NCDs compared to the UEBMI and URBMI. Furthermore, the urban-rural differences in CHE incidence slightly increased from 2012 to 2018, while the urban-rural differences in CHE intensity slightly decreased during the same period. By using the methods of Fairlie nonlinear decomposition and Blinder-Oaxaca decomposition, this research found that the household income, education and self-assessed health status of household head explained the urban-rural differences in CHE. From 2012 to 2018, the disparity explained by household income and self-assessed health status of household head increased to some extent. During the same period, the contribution of education attainment to the urban-rural differences in CHE incidence decreased, while the contribution of education attainment to the urban-rural differences in CHE intensity increased slightly. Policymakers should focus on strengthening the government’s responsibility for NRCMS, improving the household income, education attainment and health awareness of rural patients with NCDs.

## Supplementary Information

**Additional file 1: Table S1.** Out-of-pocket medical expenditure as a percentage of total medical expenditure, China, 2014–2018

## Data Availability

This study was based on a publicly available database, the China Family Panel Studies (CFPS), which was conducted by the Institute of Social Science Survey (ISSS) of Peking University every 2 y from 2010 to 2018. This database was funded by Peking University and the Chinese National Natural Foundation. The original dataset can be obtained from the official website (see link: https://isss.pku.edu.cn/cfps/download/login). The datasets used and/or analyzed during the current study are not publicly available for confidentiality reasons, but can be obtained from the corresponding author upon reasonable request.

## Abbreviations

CFPS: China Family Panel Studies
CHE: Catastrophic health expenditure
ISSS: Institute of Social Science Survey
MPO: Mean positive overshoot
NCDs: Chronic non-communicable diseases
NRCMS: New Rural Cooperative Medical Scheme
OOP: Out-of-pocket
PPS: Probability-proportional-to-scale
SDGs: Sustainable Development Goals
SMI: Supplementary Medical Insurance
UEBMI: Urban Employee Basic Medical Insurance
URBMI: Urban Residents Basic Medical Insurance
