# Supplementary material for: Urban-rural differences in catastrophic health expenditure among households with chronic non-communicable disease patients: evidence from China family panel studies
Source: BMC Public Health. 2021 May 6;21:874. doi: 10.1186/s12889-021-10887-6 (PMC8101026; doi:10.1186/s12889-021-10887-6)
Supplement: Supplementary file 1 — Additional file 1: Table S1. Out-of-pocket medical expenditure as a percentage of total medical expenditure, China, 2014–2018 [file 12889_2021_10887_MOESM1_ESM.docx]

**Supplemental Table 1.** Out-of-pocket medical expenditure as a percentage of total medical expenditure, China, 2014-2018

|  | 2014 | |  | 2016 | |  | 2018 | |
| --- | --- | --- | --- | --- | --- | --- | --- | --- |
|  | Urban | Rural |  | Urban | Rural |  | Urban | Rural |
| UEBMI | 43.48 | 47.83 |  | 50.22 | 60.81 |  | 43.34 | 59.68 |
| URBMI | 61.01 | 61.68 |  | 49.32 | 51.41 |  | 40.93 | 52.60 |
| NRCMS | 67.62 | 72.82 |  | 67.98 | 72.04 |  | 67.59 | 66.41 |
